# Effectiveness of indacaterol/glycopyrronium/mometasone for refractory asthmatic cough after switching from inhaled corticosteroid/long-acting β_2_-agonist therapy

**DOI:** 10.1016/j.jacig.2025.100567

**Published:** 2025-09-08

**Authors:** Akio Niimi, Hiroyuki Ohbayashi, Hitoshi Asamoto, Tadashi Kamei, Arihiro Kiyosue, Yasushi Fukushima, Hirofumi Matsuoka, Naoki Miyao, Takao Tochigi, Eiji Yamagata, Toshinaga Tsuji, Toshiaki Mikami, Junpei Saito, Yoshihiro Kanemitsu, Tomoko Tajiri, Johsuke Hara

**Affiliations:** a Department of Respiratory Medicine, Allergy and Clinical Immunology, Graduate School of Medical Sciences, Nagoya City University, Aichi, Japan; b Tohno Chuo Clinic, Gifu, Japan; c Asamoto Internal Medicine Clinic, Kyoto, Japan; d Kamei Internal Medicine and Respiratory Clinic, Kagawa, Japan; e Tokyo-Eki Center-Building Clinic, Tokyo, Japan; f Fukuwa Clinic, Tokyo, Japan; g Matsuoka Solairo Clinic, Kobe, Japan; h Department of Respiratory Medicine, Nippon Kokan Hospital, Kanagawa, Japan; i Tochigi Takao Clinic, Kagoshima, Japan; j Yamagata Clinic, Oita, Japan; k Division of Medical Affairs, Novartis Pharma, Tokyo, Japan; l Department of Pulmonary Medicine, School of Medicine, Fukushima Medical University, Fukushima, Japan; m Department of Respiratory Medicine, Kanazawa University Hospital, Kanazawa, Japan

**Keywords:** Asthma, indacaterol/glycopyrronium/mometasone, cough, ICS/LABA, eosinophils, neutrophils, fractional exhaled nitric oxide (Feno)

## Abstract

**Background:**

Cough is a major symptom in poorly or partially controlled asthma, which persists despite treatment, thus impairing quality of life.

**Objectives:**

The primary objective was to demonstrate the superiority of medium-dose indacaterol, glycopyrronium, and mometasone furoate (IND/GLY/MF) over high-dose inhaled corticosteroids (ICS)/long-acting β_2_-agonists (LABA) in adult asthma patients with cough refractory to medium-dose ICS/LABA.

**Methods:**

In this multicenter, randomized, open-label, parallel-group study, 118 patients were randomized to receive either IND/GLY/MF or high-dose ICS/LABA (fluticasone/vilanterol [FF/VI] or budesonide/formoterol [BUD/FM]) for 8 weeks. Efficacy was measured by the Japanese version of the Leicester Cough Questionnaire at week 8. Additional analyses included a visual analog scale, the 6-item Asthma Control Questionnaire, respiratory function, fractional exhaled nitric oxide, and blood eosinophil/neutrophil measurements. Registration: Public clinical trials registry as study jRCTs04122000.

**Results:**

The change from baseline to week 8 in the total questionnaire score was 1.99 ± 3.48 in the IND/GLY/MF group and 2.50 ± 4.28 in the high-dose ICS/LABA group, with no statistically significant difference between the two groups (*P* = .5037). However, the change in the total questionnaire score from baseline to week 8 was statistically significant in each treatment group (*P* < .0001 in the IND/GLY/MF group, *P* = .0242 in the FF/VI comparator group, and *P* = .0012 in the BUD/FM comparator group). Fractional exhaled nitric oxide and eosinophil counts significantly decreased in the high-dose ICS/LABA group, while neutrophil counts significantly fell in the IND/GLY/MF group at week 8, by between-group comparison.

**Conclusion:**

Symptoms improved comparably between medium-dose IND/GLY/MF and high-dose ICS/LABA combinations, although no superiority could be demonstrated. Medium-dose IND/GLY/MF may be an alternative to high-dose ICS/LABA.

## Graphical abstract

**Figure:**
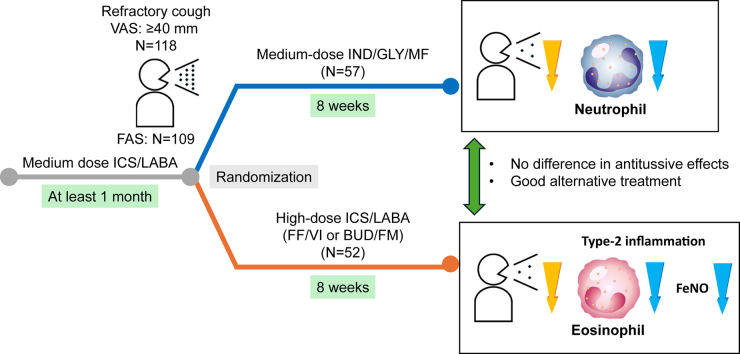

According to the World Health Organization, asthma affected approximately 262 million people in 2019 and caused 455,000 deaths worldwide.^1^ Among various symptoms of asthma, including cough, sputum, wheezing, chest tightness, and shortness of breath, cough has the greatest impact and highest burden for patients, limiting their daily activities.^2^ An observational study conducted in Japan revealed that more than 40% of asthma patients treated by allergologists and respiratory specialists still have cough symptoms, despite standard treatment including inhaled corticosteroid (ICS) therapy.^3^ Furthermore, studies have shown that increased cough symptoms are associated with progression of asthma severity and higher incidence of exacerbations, poor prognosis, and decreased quality of life.^4^, ^5^, ^6^, ^7^ Thus, cough is a key to and an important factor in asthma management.^8^

The American College of Chest Physicians’ CHEST Guideline and Expert Panel Report,^9^ based on systematic reviews of randomized controlled trials and observational studies, recommend ICS as treatment for cough in asthma patients. For persistent cough, it is recommended to increase the ICS dose or to add leukotriene receptor antagonists or β_2_-agonists to the treatment regimen. However, there are a considerable number of asthma patients with persistent daytime (44.5%) and nighttime (39.1%) cough despite appropriate ICS treatment.^3^ Despite treatment with ICS in combination with long-acting β_2_-agonists (LABA), the frequency of uncontrolled asthma is still high at 53.7% according to the 2018 Japanese Guidelines for Asthma and 36.3% according to the 2019 Global Initiative for Asthma definitions.^10^

The 2021 Asthma Prevention and Management Guidelines published by the Japanese Society of Allergology recommends the addition of long-acting muscarinic antagonists (LAMAs) or dose increase of ICS as the next steps for patients with poorly controlled asthma while receiving medium-dose ICS/LABA.^11^^,^^12^ Tiotropium, a LAMA, has been shown to significantly improve cough as assessed by the visual analog scale (VAS), Leicester Cough Questionnaire (LCQ), and Asthma Control Test in asthma patients who had persistent cough symptoms despite ICS/LABA therapy.^13^^,^^14^ The addition of tiotropium to ICS/LABA significantly improved capsaicin cough receptor sensitivity, showing a correlation between changes in cough VAS and capsaicin cough receptor sensitivity.^14^ Intravenous administration of glycopyrronium bromide, another LAMA, inhibited cough during endoscopic submucosal dissection of upper gastrointestinal neoplasia.^15^

The LABA/LAMA/ICS combination of indacaterol, glycopyrronium, and mometasone furoate (IND/GLY/MF) has previously been compared to ICS/LABA combinations in patients with uncontrolled asthma.^16^^,^^17^ High and medium doses of IND/GLY/MF demonstrated superiority versus high- and medium-dose IND/MF and high-dose salmeterol/fluticasone with regard to improvements in trough forced expiratory volume in 1 second (FEV_1_)^18^ and noninferiority versus high-dose salmeterol/fluticasone plus tiotropium with regard to trough FEV_1_, and peak expiratory flow in the morning and evening.^19^

We have previously described the design of the REACH study, in which we treated Japanese asthma patients with refractory cough despite medium-dose ICS/LABA treatment with a fixed medium dose of IND/GLY/MF compared to two different high-dose combinations of ICS/LABA.^20^ Ours is the first study to evaluate the effectiveness of IND/GLY/MF versus ICS/LABA in asthma patients who have refractory cough that is resistant to treatment with medium-dose ICS/LABA. Here we present the results of this study, addressing both the antitussive and anti-inflammatory effects of the treatments.

## Methods

The study was conducted from October 2022 to September 2023 in compliance with the ethical principles stipulated in the Declaration of Helsinki, the Japanese Clinical Trials Act and related notifications, and the research protocol. It was approved by the certified review board of Nagoya City University (approval 2022A002). Key study information (eg, study design and recruitment information) was registered at the Japan Registry of Clinical Trials (identifier jRCTs041220003).

This multicenter, randomized, open-label, parallel-group study was conducted at 12 study centers in Japan, involving 118 adult asthma patients with refractory cough despite treatment with medium doses of ICS/LABA for at least 1 month. Asthma patients with refractory cough were defined as those who still had a cough VAS of ≥40 mm while awake despite moderate-dose ICS/LABA treatment for ≥1 month.^21^ Details of the study design have been reported previously.^20^

All patients provided informed consent to participate in the study and were informed about their right to discontinue the study at any time without penalty or loss of benefits. After a 2-week screening period, patients were randomized into one of the 3 treatment groups in a 2:1:1 ratio to receive open-label daily inhalations of either medium-dose (150 μg/50 μg/80 μg) IND/GLY/MF, or one of the two formulations of high-dose ICS/LABA as control for 8 weeks. In control group 1, patients received 25 μg vilanterol and 200 μg fluticasone furoate (FF/VI); in control group 2, they received 1280 μg budesonide and 36 μg formoterol fumarate hydrate (BUD/FM). IND/GLY/MF were compared to both high-dose ICS/LABA groups combined as well as to each of the ICS/LABA groups.

The primary end point of the study was the change from baseline in the Japanese version of the LCQ (J-LCQ) score^22^^,^^23^ after 8 weeks of treatment with IND/GLY/MF medium-dose versus high-dose ICS/LABA (FF/VI or BUD/FM). The minimal clinically important difference (MCID) was defined as 1.3 in chronic cough.^24^

The secondary end points included cough VAS assessments after 8 weeks while waking or sleeping, and the percentage of patients with MCID ≥ 15 mm improvement on the VAS^13^^,^^25^ from baseline or an absolute value of <40 mm in cough VAS.

Other tests after 8 weeks included spirometry of respiratory function parameters, fractional exhaled nitric oxide (Feno), blood eosinophil and neutrophil counts, and administration of the Six-item Asthma Control Questionnaire (ACQ-6) and the Cough and Sputum Assessment Questionnaire (CASA-Q).

For a capsaicin cough receptor sensitivity test, capsaicin solution was inhaled to evaluate the sensitivity of cough receptors of patients, to evaluate the concentration of capsaicin causing 2 (C2) or 5 (C5) coughs during the 1-minute period between each dose. A VitaloJAK cough monitor was used to record the frequency of cough in patients for 24 hours. Both of these tests were done in 6 patients in the IND/GLY/MF group and 1 patient in the ICS/LABA group, thus only yielding data for a single (the first) arm of the study.

Safety in this study was assessed by analyzing the number and percentage of patients with adverse events (AEs) and adverse drug reactions in the IND/GLY/MF group and the ICS/LABA group.

Statistical analysis was performed by SAS v9.4 software (SAS Institute). To demonstrate the superiority of IND/GLY/MF medium-dose over high-dose ICS/LABA (FF/VI or BUD/FM), the target sample size was calculated to be 212 patients by RStudio v1.1.456 software (rstudio.com) with the MKpower package. The calculation was based on a dropout rate of 10% and the achievement of at least 80% power (with multiplicity adjustment) for the primary end point (J-LCQ score) and major secondary end point (cough VAS score).^20^

For the primary end point, a closed testing procedure and Bonferroni method were used to control the family-wise type I error at the 1-sided α level of 0.025 with the following steps:

1. Compare IND/GLY/MF with ICS/LABA (FF/VI, BUD/FM) for J-LCQ.
2a. Compare IND/GLY/MF with ICS/LABA (FF/VI, BUD/FM) for cough VAS.
2b. Compare IND/GLY/MF with FF/VI and BUD/FM for J-LCQ.
3. Compare IND/GLY/MF with FF/VI and BUD/FM for cough VAS.

The testing method for steps 1 and 2a was the Welch *t* test; the testing method in steps 2b and 3 was the Dunnett test based on the IND/GLY/MF group. In step 1, IND/GLY/MF was considered superior at *P* < .025. In steps 2a, 2b, and 3, superiority of IND/GLY/MF was established at *P* < .0125. If no superiority could be established at any step, the superiority verification was completed and was not confirmed at subsequent steps, even though the analyses in each step were performed.

The lower number of recruited patients did not affect the goal to detect significant differences at all steps, as high power was set by reducing the consumption of the α level in a step-by-step manner, which maintained a power of at least 80% at each analysis step (96.8% for step 1, 97.7% for step 2a, 81.3% for step 2b, and 85.4% for step 3).

For other analyses, the Welch test was performed to compare the mean change from baseline between the groups, and a chi-square test was performed to compare the percentage between the groups. The significance level for the Welch *t* test was 2.5% for 1-sided test and 5% for 2-sided test; and that of the chi-square test was 5%. No multiplicity adjustment was performed. One-sample *t* tests were performed for the change of parameters from baseline to week 4 or week 8.

## Results

**Fig 1:**
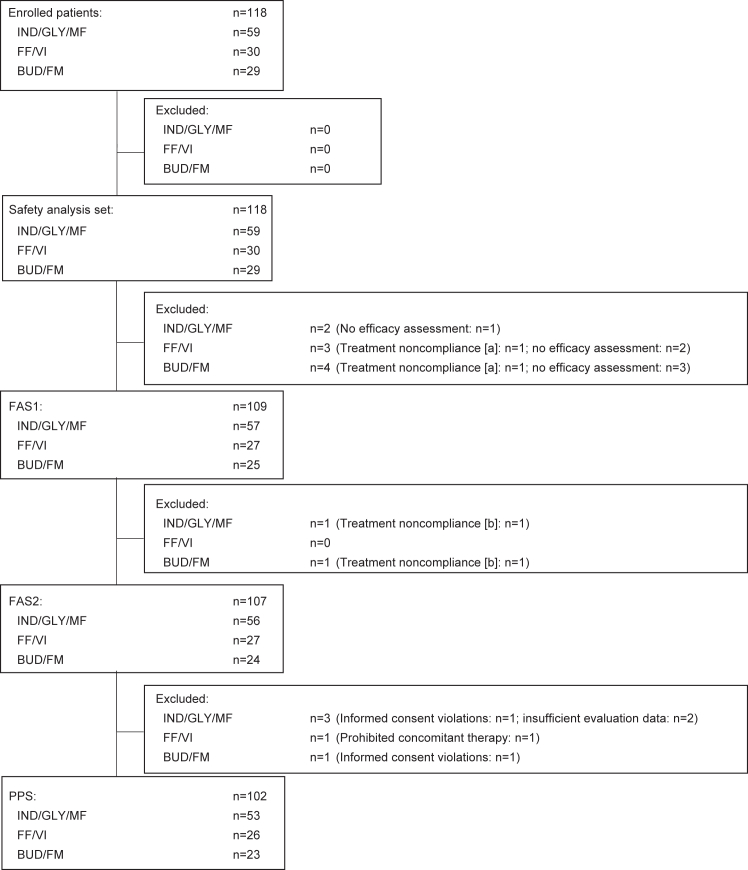
Patient enrollment. **(A)** In FAS1, patients whose treatment noncompliance was discovered during protocol treatment and whose treatment was discontinued were excluded. **(B)** In FAS2, patients whose treatment noncompliance was discovered during protocol treatment but who then subsequently continued that treatment were also excluded. *PPS,* Per-protocol set.

Of the 118 patients who were enrolled onto the study and received study medication, 109 had at least one evaluation and were included in full analysis set 1 (FAS1). In FAS2, two patients were excluded who had a treatment compliance violation, and in the per-protocol set, 7 patients were excluded who had any treatment compliance violation, missing assessments, or other significant protocol violations (Fig 1). Of the 109 patients in FAS1, 57 were receiving medium-dose IND/GLY/MF, 52 patients were in the high-dose ICS/LABA groups with 27 patients receiving FF/VI, and 25 patients were receiving BUD/FM.

The patient demographics and baseline characteristics are shown in Table I. Overall, the majority of patients were female (68.4% in the IND/GLY/MF group and 65.4% in the ICS/LABA group), and the mean age was 50.8 years in the IND/GLY/MF group and 50.4 years in the ICS/LABA group. All patients were recruited in Japan; however, no data about race and ethnicity were collected. There were no notable differences between the IND/GLY/MF group and the ICS/LABA group in any of the baseline parameters.

**Table I:** Patient demographic and other baseline characteristics

Characteristic	Variable	IND/GLY/MF (no.)	Total ICS/LABA (no.)	FF/VI (no.)	BUD/FM (no.)
No. of subjects		57	52	27	25
Sex (no.)	Male	31.6% (18)	34.6% (18)	37.0% (10)	32.0% (8)
	Female	68.4% (39)	65.4% (34)	63.0% (17)	68.0% (17)
Age (years)	Mean ± SD (no.)	50.8 ± 15.7 (57)	50.4 ± 14.2 (52)	50.4 ± 13.2 (27)	50.3 ± 15.6 (25)
	Min, median, max	21, 51.0, 78	22, 51.0, 75	22, 51.0, 74	22, 53.0, 75
Height (cm)	Mean ± SD (no.)	160.71 ± 9.49 (57)	161.37 ± 9.24 (52)	162.39 ± 8.09 (27)	160.27 ± 10.39 (25)
	Min, median, max	140.5, 162.00, 180.0	141.6, 161.00, 179.9	148.7, 161.40, 176.7	141.6, 160.00, 179.9
Weight (kg)	Mean ± SD (no.)	61.26 ± 12.19 (57)	60.56 ± 12.74 (52)	59.31 ± 12.36 (27)	61.92 ± 13.24 (25)
	Min, median, max	38.2, 59.50, 83.0	38.3, 59.70, 97.5	42.0, 57.60, 90.3	38.3, 60.40, 97.5
Body mass index (kg/m^2^)	Mean ± SD (no.)	23.67 ± 4.04 (57)	23.25 ± 4.49 (52)	22.47 ± 4.26 (27)	24.08 ± 4.67 (25)
	Min, median, max	15.7, 23.90, 33.0	16.2, 22.75, 38.3	16.2, 21.20, 32.3	18.3, 23.10, 38.3
Smoking history	No	87.7% (50)	80.8% (42)	85.2% (23)	76.0% (19)
	Yes	12.3% (7)	19.2% (10)	14.8% (4)	24.0% (6)
Complications	No	15.8% (9)	21.2% (11)	22.2% (6)	20.0% (5)
	Yes	84.2% (48)	78.8% (41)	77.8% (21)	80.0% (20)
Types of complications	Allergic rhinitis	40.7% (24)	44.1% (26)	59.3% (16)	40.0% (10)
	Atopic dermatitis	10.5% (6)	9.6% (5)	7.4% (2)	12.0% (3)
Medical history	No	73.7% (42)	75.0% (39)	66.7% (18)	84.0% (21)
	Yes	26.3% (15)	25.0% (13)	33.3% (9)	16.0% (4)
ICS/LABA	FF/VI	77.2% (44)	80.8% (42)	77.8% (21)	84.0% (21)
	BUD/FM	22.8% (13)	19.2% (10)	22.2% (6)	16.0% (4)
Duration of asthma (years)∗	Mean ± SD (no.)	8.28 ± 9.99 (57)	10.22 ± 10.59 (52)	12.29 ± 11.82 (27)	8.00 ± 8.76 (25)
	Min, median, max	0.3, 4.92, 46.1	0.3, 6.42, 50.9	0.3, 10.28, 50.9	0.4, 5.62, 38.2
Severity of asthma	Mild persistent	12.3% (7)	25.0% (13)	33.3% (9)	16.0% (4)
	Moderate persistent	86.0% (49)	67.3% (35)	63.0% (17)	72.0% (18)
	Severe persistent	1.8% (1)	7.7% (4)	3.7% (1)	12.0% (3)
	Most severe persistent	0	0	0	0
History of childhood asthma	No	94.7% (54)	76.9% (40)	70.4% (19)	84.0% (21)
	Yes	5.3% (3)	23.1% (12)	29.6% (8)	16.0% (4)
Family history of asthma	No	68.4% (39)	61.5% (32)	63.0% (17)	60.0% (15)
	Yes	31.6% (18)	38.5% (20)	37.0% (10)	40.0% (10)
Concomitant medications for asthma	No	54.4% (31)	59.6% (31)	55.6% (15)	64.0% (16)
	Yes	45.6% (26)	40.4% (21)	44.4% (12)	36.0% (9)
J-LCQ total score	Mean ± SD	15.12 ± 2.80 (57)	15.00 ± 3.15 (52)	15.33 ± 2.85 (27)	14.64 ± 3.47 (25)
	Min, median, max	6.0, 15.52, 20.0	6.9, 15.25, 21.0	6.9, 15.63, 21.0	7.4, 14.70, 20.6
Cough VAS score while awake (mm)	Mean ± SD	55.6 ± 9.9 (57)	58.4 ± 11.9 (52)	56.5 ± 10.8 (27)	60.4 ± 12.9 (25)
	Min, median, max	40, 53.0, 78	41, 59.0, 84	43, 57.0, 83	41, 60.0, 84
Cough VAS score while asleep (mm)	Mean ± SD	39.3 ± 24.9 (57)	42.8 ± 26.3 (52)	46.0 ± 22.9 (27)	39.4 ± 29.7 (25)
	Min, median, max	0, 51.0, 79	0, 48.0, 87	3, 51.0, 86	0, 43.0, 87
Capsaicin (C2) cough receptor sensitivity	Mean ± SD	1.708 ± 1.716 (6)	0.980 ± NC (1)	0.980 ± NC (1)	(0)
	Min, median, max	0.49, 0.915, 4.88	—	—	—
Capsaicin (C5) cough receptor sensitivity	Mean ± SD	4.250 ± 4.351 (6)	0.980 ± NC (1)	0.980 ± NC (1)	(0)
	Min, median, max	0.49, 2.440, 9.76	—	—	—
Cough frequency (times per hour) (24 hours)†	Mean ± SD	23.69 ± 16.11 (6)	7.42 ± NC (1)	7.42 ± NC (1)	(0)
	Min, median, max	4.0, 24.81, 45.3	—	—	—
Cough frequency while awake (times per hour)†	Mean ± SD	23.91 ± 13.98 (6)	9.75 ± NC (1)	9.75 ± NC (1)	(0)
	Min, median, max	5.0, 27.56, 40.0	—	—	—
Cough frequency while asleep (times per hour)†	Mean ± SD	23.25 ± 26.96 (6)	2.75 ± NC (1)	2.75 ± NC (1)	(0)
	Min, median, max	1.9, 15.81, 74.4	—	—	—
FEV_1_ (L)	Mean ± SD	2.547 ± 0.791 (57)	2.665 ± 0.841 (52)	2.709 ± 0.853 (27)	2.617 ± 0.842 (25)
	Min, median, max	1.17, 2.450, 4.64	0.95, 2.675, 4.82	0.95, 2.780, 4.23	1.39, 2.370, 4.82
FVC (L)	Mean ± SD	3.137 ± 0.819 (57)	3.300 ± 0.842 (52)	3.346 ± 0.775 (27)	3.251 ± 0.922 (25)
	Min, median, max	1.66, 3.040, 5.19	1.67, 3.135, 5.80	1.67, 3.420, 4.80	2.10, 3.050, 5.80
FEF_25-75%_ (L/s)	Mean ± SD	2.75 ± 1.46 (57)	2.82 ± 1.44 (52)	2.92 ± 1.43 (27)	2.71 ± 1.46 (25)
	Min, median, max	0.4, 2.80, 7.9	0.3, 2.70, 5.7	0.3, 3.00, 5.4	0.7, 2.60, 5.7
%FEV_1_ (%)	Mean ± SD	95.52 ± 17.24 (57)	97.54 ± 19.66 (52)	97.63 ± 21.92 (27)	97.45 ± 17.34 (25)
	Min, median, max	55.6, 98.65, 131.8	29.5, 99.67, 131.4	29.5, 99.04, 131.4	60.3, 99.95, 126.3
FEV_1_/FVC (%)	Mean ± SD	80.71 ± 9.34 (57)	79.89 ± 10.74 (52)	79.69 ± 12.44 (27)	80.10 ± 8.78 (25)
	Min, median, max	48.4, 82.07, 98.9	38.5, 82.78, 95.1	38.5, 83.74, 92.4	63.3, 80.60, 95.1
Feno (ppb)	Mean ± SD	25.2 ± 25.4 (57)	31.1 ± 39.1 (52)	31.4 ± 26.0 (27)	30.7 ± 50.1 (25)
	Min, median, max	5, 18.0, 177	5, 19.0, 265	6, 19.0, 92	5, 19.0, 265
Eosinophils (/μL)	Mean ± SD	231.3 ± 221.2 (56)	292.3 ± 259.9 (52)	327.4 ± 298.2 (27)	254.4 ± 210.4 (25)
	Min, median, max	0, 130.0, 900	40, 195.0, 1240	70, 190.0, 1240	40, 200.0, 880
Neutrophils (/μL)	Mean ± SD	4361.4 ± 1532.5 (56)	3822.9 ± 1345.1 (52)	3520.0 ± 1258.5 (27)	4150.0 ± 1383.5 (25)
	Min, median, max	1630, 4210.0, 9420	1430, 3460.0, 8760	1430, 3240.0, 7050	2250, 4020.0, 8760
ACQ-6 (total score)	Mean ± SD	1.40 ± 0.53 (57)	1.48 ± 0.84 (52)	1.44 ± 0.82 (27)	1.53 ± 0.88 (25)
	Min, median, max	0.2, 1.33, 2.5	0.0, 1.50, 3.7	0.0, 1.50, 3.7	0.2, 1.50, 3.0
CASA-Q (total score)	Mean ± SD	75.7 ± 12.6 (57)	72.9 ± 14.3 (52)	72.5 ± 13.2 (27)	73.4 ± 15.6 (25)
	Min, median, max	44, 77.5, 100	38, 76.9, 100	38, 75.0, 100	45, 77.5, 95

*NC,* Not calculable.

∗ Duration of asthma = [(date of informed consent − date of diagnosis of asthma)/365.25]. When date of diagnosis of asthma is unknown (month and day), it is calculated as January 1.

† Cough frequency by VitaloJAK cough monitor.

### J-LCQ score

**Fig 2:**
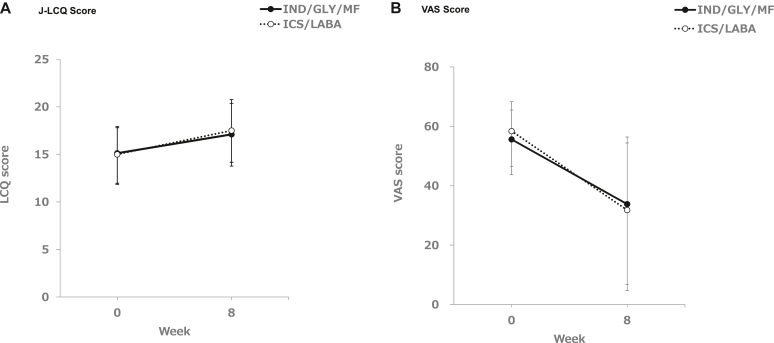
Change (±SD) of J-LCQ score and cough as assessed by VAS from baseline to week 8.

In analysis step 1, the change from baseline in the J-LCQ total score at week 8 of treatment was 1.99 ± 3.48 in the IND/GLY/MF group and 2.50 ± 4.28 in the ICS/LABA group, with no statistically significant difference between the groups, failing to demonstrate superiority (*P* = .5037). Fig 2, *A,* shows the change in J-LCQ score in the two treatment groups.

The change from baseline in the total score of the J-LCQ at week 8 was 2.11 ± 4.59 in the FF/VI comparator group and 2.91 ± 3.97 in the BUD/FM comparator group, with no statistically significant difference between the IND/GLY/MF group and either of the ICS/LABA groups (*P* = .9882 and *P* = .5343, respectively).

However, the change from baseline to week 8 was statistically significant in each treatment group (*P* < .0001in the IND/GLY/MF group, *P* = .0242 in the FF/VI comparator group, and *P* = .0012 in the BUD/FM comparator group).

The proportion of patients who experienced MCID ≥ 1.3 in J-LCQ was 40.4% in the IND/GLY/MF group and 61.5% in the ICS/LABA group at week 4, and 52.7% in the IND/GLY/MF group and 57.7% in the ICS/LABA group at week 8 (Table II). The proportion of patients who showed significant improvement in the ICS/LABA group was higher compared to the IND/GLY/MF group at week 4, but there was no significant difference between the two groups at week 8 (week 4 *P* = .0271, week 8 *P* = .6058).

**Table II:** J-LCQ score showing proportion of patients with MCID ≥ 1.3 from baseline

Week	Regimen	MCID in J-LCQ score (no.)		*P* value
		<1.3	≥1.3	
4	IND/GLY/MF	59.6% (34)	40.4% (23)	.0271
	ICS/LABA	38.5% (20)	61.5% (32)	
8	IND/GLY/MF	47.3% (26)	52.7% (29)	.6058
	ICS/LABA	42.3% (22)	57.7% (30)	

*P* value calculated by chi-square test.

### Cough VAS

The overall change from baseline to week 8 in the cough VAS score is presented in Fig 2, *B.* The daytime VAS scores while the patients were awake and the nighttime VAS scores when they were sleeping all changed significantly from baseline to week 8 in every treatment group; however, there was no statistically significant difference between the groups (Table III).

**Table III:** Cough as assessed by VAS

Parameter	Week	Variable	Treatment groups	Mean ± SD (no.)	*P*, Welch *t* test∗	*P*, 1-sample *t* test†
Cough VAS score while awake (mm)	0	Observed value	IND/GLY/MF	55.6 ± 9.9 (57)		
			ICS/LABA	58.4 ± 11.9 (52)		
			FF/VI	56.5 ± 10.8 (27)		
			BUD/FM	60.4 ± 12.9 (25)		
	4	Observed value	IND/GLY/MF	42.5 ± 23.2 (57)		
			ICS/LABA	36.1 ± 25.4 (52)		
		Change	IND/GLY/MF	−13.0 ± 23.6 (57)		
			ICS/LABA	−22.3 ± 24.1 (52)	.0448	
	8	Observed value	IND/GLY/MF	33.8 ± 22.6 (55)		
			ICS/LABA	31.8 ± 27.1 (52)		
			FF/VI	33.1 ± 25.8 (27)		
			BUD/FM	30.3 ± 28.9 (25)		
		Change	IND/GLY/MF	−21.4 ± 22.4 (55)		<.0001
			ICS/LABA	−26.6 ± 28.5 (52)	.2986	
			FF/VI	−23.4 ± 27.9 (27)		.0002
			BUD/FM	−30.1 ± 29.4 (25)		<.0001
Cough VAS score while asleep (mm)	0	Observed value	IND/GLY/MF	39.3 ± 24.9 (57)		
			ICS/LABA	42.8 ± 26.3 (52)		
			FF/VI	46.0 ± 22.9 (27)		
			BUD/FM	39.4 ± 29.7 (25)		
	4	Observed value	IND/GLY/MF	32.1 ± 26.4 (57)		
			ICS/LABA	31.6 ± 27.6 (52)		
		Change	IND/GLY/MF	−7.2 ± 24.8 (57)		
			ICS/LABA	−11.2 ± 23.5 (52)	.3891	
	8	Observed value	IND/GLY/MF	28.0 ± 25.5 (55)		
			ICS/LABA	25.2 ± 24.4 (52)		
			FF/VI	26.4 ± 23.1 (27)		
			BUD/FM	23.9 ± 26.0 (25)		
		Change	IND/GLY/MF	−11.6 ± 29.0 (55)		.0044
			ICS/LABA	−17.6 ± 23.2 (52)	.2388	
			FF/VI	−19.6 ± 24.7 (27)		.0003
			BUD/FM	−15.5 ± 21.6 (25)		.0015

∗ Comparison between IND/GLY/MF and ICS/LABA.

† Comparison between baseline and week 8.

### Other secondary end points

Results of the respiratory function tests (FEV_1_, forced vital capacity [FVC], forced expiratory flow between 25% and 75% of FVC [FEF_25-75%_], percentage predicted FEV_1_ [%FEV_1_], and FEV_1_/%FVC) at baseline and week 8 are shown in Table IV.^26^ No statistically significant differences were observed between the IND/GLY/MF group and the ICS/LABA group for FEV_1_ (*P* = .9870), FVC (*P* = .7240), FEF_25-75%_ (*P* = .4394), %FEV_1_ (*P* = .9101), or FEV_1_/%FVC (*P* = .7004), by Welch *t* test).

**Table IV:** Respiratory function test by spirometry

Parameter	Week	Variable	Treatment group	Mean ± SD (no.)	*P*, Welch *t* test∗	*P*, 1-sample *t* test†
FEV_1_ (L)	0	Observed value	IND/GLY/MF	2.547 ± 0.791 (57)		
			ICS/LABA	2.665 ± 0.841 (52)		
	8	Observed value	IND/GLY/MF	2.598 ± 0.774 (54)		
			ICS/LABA	2.709 ± 0.816 (52)		
		Change	IND/GLY/MF	0.044 ± 0.181 (54)		
			ICS/LABA	0.044 ± 0.147 (52)	.9870	
FEV_1_ CC (mL)	0	Observed value	IND/GLY/MF	2546.7 ± 791.0 (57)		
			FF/VI	2709.3 ± 853.1 (27)		
			BUD/FM	2616.8 ± 842.5 (25)		
	8	Observed value	IND/GLY/MF	2598.1 ± 774.0 (54)		
			FF/VI	2746.7 ± 807.2 (27)		
			BUD/FM	2668.0 ± 839.4 (25)		
		Change	IND/GLY/MF	43.5 ± 180.5 (54)		.0822
			FF/VI	37.4 ± 133.2 (27)		.1566
			BUD/FM	51.2 ± 162.3 (25)		.1278
FVC (L)	0	Observed value	IND/GLY/MF	3.137 ± 0.819 (57)		
			ICS/LABA	3.300 ± 0.842 (52)		
	8	Observed value	IND/GLY/MF	3.182 ± 0.801 (54)		
			ICS/LABA	3.353 ± 0.808 (52)		
		Change	IND/GLY/MF	0.040 ± 0.194 (54)		
			ICS/LABA	0.052 ± 0.174 (52)	.7240	
FVC CC (mL)	0	Observed value	IND/GLY/MF	3137.0 ± 818.9 (57)		
			FF/VI	3345.9 ± 775.3 (27)		
			BUD/FM	3251.2 ± 922.4 (25)		
	8	Observed value	IND/GLY/MF	3182.4 ± 801.2 (54)		
			FF/VI	3393.0 ± 753.4 (27)		
			BUD/FM	3309.2 ± 876.4 (25)		
		Change	IND/GLY/MF	39.6 ± 194.2 (54)		.1396
			FF/VI	47.0 ± 158.4 (27)		.1349
			BUD/FM	58.0 ± 193.1 (25)		.1462
FEF_25-75%_ (L/s)	0	Observed value	IND/GLY/MF	2.75 ± 1.46 (57)		
			ICS/LABA	2.82 ± 1.44 (52)		
	8	Observed value	IND/GLY/MF	2.89 ± 1.47 (54)		
			ICS/LABA	3.13 ± 2.47 (52)		
		Change	IND/GLY/MF	0.12 ± 0.44 (54)		
			ICS/LABA	0.31 ± 1.70 (52)	.4394	
FEF_25-75%_ CC (mL)	0	Observed value	IND/GLY/MF	2.75 ± 1.46 (57)		
			FF/VI	2.92 ± 1.43 (27)		
			BUD/FM	2.71 ± 1.46 (25)		
	8	Observed value	IND/GLY/MF	2.89 ± 1.47 (54)		
			FF/VI	3.43 ± 3.10 (27)		
			BUD/FM	2.80 ± 1.52 (25)		
		Change	IND/GLY/MF	0.12 ± 0.44 (54)		.0489
			FF/VI	0.51 ± 2.31 (27)		.2571
			BUD/FM	0.09 ± 0.51 (25)		.3970
%FEV1‡	0	Observed value	IND/GLY/MF	95.52 ± 17.24 (57)		
			ICS/LABA	97.54 ± 19.66 (52)		
			FF/VI	97.630 ± 21.924 (27)		
			BUD/FM	97.452 ± 17.342 (25)		
	8	Observed value	IND/GLY/MF	98.33 ± 17.57 (54)		
			ICS/LABA	99.53 ± 19.47 (52)		
			FF/VI	99.658 ± 21.209 (27)		
			BUD/FM	99.402 ± 17.846 (25)		
		Change	IND/GLY/MF	2.14 ± 6.79 (54)		.0246
			ICS/LABA	1.99 ± 6.44 (52)	.9101	
			FF/VI	2.028 ± 6.584 (27)		.1216
			BUD/FM	1.950 ± 6.410 (25)		.1413
FEV_1_/%FVC	0	Observed value	IND/GLY/MF	80.71 ± 9.34 (57)		
			ICS/LABA	79.89 ± 10.74 (52)		
			FF/VI	79.686 ± 12.443 (27)		
			BUD/FM	80.101 ± 8.782 (25)		
	8	Observed value	IND/GLY/MF	81.24 ± 8.69 (54)		
			ICS/LABA	80.05 ± 10.51 (52)		
			FF/VI	79.954 ± 11.485 (27)		
			BUD/FM	80.146 ± 9.584 (25)		
		Change	IND/GLY/MF	0.42 ± 3.77 (54)		.4206
			ICS/LABA	0.16 ± 3.04 (52)	.7004	
			FF/VI	0.268 ± 3.157 (27)		.6627
			BUD/FM	0.044 ± 2.965 (25)		.9409

*CC,* Closing capacity.

∗ Comparison between IND/GLY/MF and ICS/LABA.

† Comparison between baseline and week 8.

‡ Percentage of reference value calculated by LMS method according to Kubota et al, 2014.

Results of ACQ-6 score, CASA-Q score, Feno, and eosinophil and neutrophil counts are shown in Table V. No significant difference between the IND/GLY/MF group and the ICS/LABA group at week 8 were seen for ACQ-6 score (*P* = .3276) and CASA-Q score (*P* = .3685). There was a significant improvement for Feno in the ICS/LABA group compared to the IND/GLY/MF group (*P* = .0230; see Fig E1 in this article’s Online Repository available at www.jaci-global.org) and a significant decrease in blood eosinophil count in the ICS/LABA group compared to the IND/GLY/MF group (*P* = .0101; see Fig E2 in the Online Repository). In contrast, blood neutrophil count significantly decreased in the IND/GLY/MF group compared to the ICS/LABA group (*P* = .0141; see Fig E3 in the Online Repository).

**Table V:** Change of study test results from baseline to week 8

Parameter	Week	Variable	Treatment group	Mean ± SD (no.)	*P*, Welch *t* test∗	*P*, 1-sample *t* test†
ACQ-6 score	0	Observed value	IND/GLY/MF	1.398 ± 0.532 (57)		
			ICS/LABA	1.48 ± 0.84 (52)		
			FF/VI	1.444 ± 0.815 (27)		
			BUD/FM	1.526 ± 0.878 (25)		
	4	Observed value	IND/GLY/MF	1.23 ± 0.70 (57)		
			ICS/LABA	0.93 ± 0.74 (52)		
		Change	IND/GLY/MF	−0.17 ± 0.67 (57)		
			ICS/LABA	−0.56 ± 0.75 (52)	.0059	
	8	Observed value	IND/GLY/MF	0.858 ± 0.580 (55)		
			ICS/LABA	0.78 ± 0.82 (52)		
			FF/VI	0.766 ± 0.869 (27)		
			BUD/FM	0.800 ± 0.778 (25)		
		Change	IND/GLY/MF	−0.533 ± 0.685 (55)		.0001
			ICS/LABA	−0.70 ± 1.04 (52)	.3276	
			FF/VI	−0.679 ± 1.222 (27)		.0078
			BUD/FM	−0.726 ± 0.821 (25)		.0002
CASA-Q overall score	0	Observed value	IND/GLY/MF	75.7 ± 12.6 (57)		
			ICS/LABA	72.9 ± 14.3 (52)		
			FF/VI	72.454 ± 13.235 (27)		
			BUD/FM	73.400 ± 15.552 (25)		
	4	Observed value	IND/GLY/MF	79.7 ± 14.5 (57)		
			ICS/LABA	83.7 ± 13.6 (52)		
		Change	IND/GLY/MF	4.0 ± 14.3 (57)		
			ICS/LABA	10.8 ± 12.1 (52)	.0082	
	8	Observed value	IND/GLY/MF	84.3 ± 12.0 (55)		
			ICS/LABA	84.0 ± 15.6 (52)		
			FF/VI	82.685 ± 17.554 (27)		
			BUD/FM	85.350 ± 13.305 (25)		
		Change	IND/GLY/MF	8.3 ± 14.3 (55)		.0001
			ICS/LABA	11.1 ± 17.1 (52)	.3685	
			FF/VI	10.231 ± 20.144 (27)		.0139
			BUD/FM	11.950 ± 13.516 (25)		.0002
CASA-Q cough symptoms	0	Observed value	IND/GLY/MF	63.3 ± 14.9 (57)		
			ICS/LABA	55.9 ± 19.3 (52)		
			FF/VI	58.333 ± 18.199 (27)		
			BUD/FM	53.333 ± 20.412 (25)		
	4	Observed value	IND/GLY/MF	65.8 ± 19.1 (57)		
			ICS/LABA	74.0 ± 16.5 (52)		
		Change	IND/GLY/MF	2.5 ± 19.9 (57)		
			ICS/LABA	18.1 ± 20.1 (52)	.0001	
	8	Observed value	IND/GLY/MF	73.2 ± 17.3 (55)		
			ICS/LABA	73.9 ± 21.2 (52)		
			FF/VI	73.148 ± 21.225 (27)		
			BUD/FM	74.667 ± 21.582 (25)		
		Change	IND/GLY/MF	9.4 ± 19.8 (55)		.0009
			ICS/LABA	17.9 ± 26.5 (52)	.0628	
			FF/VI	14.814 ± 26.588 (27)		.0076
			BUD/FM	21.334 ± 26.580 (25)		.0005
CASA-Q cough impact	0	Observed value	IND/GLY/MF	77.5 ± 14.4 (57)		
			ICS/LABA	72.7 ± 17.0 (52)		
			FF/VI	72.457 ± 16.308 (27)		
			BUD/FM	73.002 ± 18.129 (25)		
	4	Observed value	IND/GLY/MF	82.3 ± 16.2 (57)		
			ICS/LABA	85.6 ± 14.4 (52)		
		Change	IND/GLY/MF	4.9 ± 15.7 (57)		
			ICS/LABA	12.9 ± 14.8 (52)	.0073	
	8	Observed value	IND/GLY/MF	87.3 ± 13.3 (55)		
			ICS/LABA	85.8 ± 18.2 (52)		
			FF/VI	84.724 ± 20.589 (27)		
			BUD/FM	87.002 ± 15.590 (25)		
		Change	IND/GLY/MF	9.5 ± 16.1 (55)		.0001
			ICS/LABA	13.1 ± 22.3 (52)	.3494	
			FF/VI	12.267 ± 25.754 (27)		.0202
			BUD/FM	14.000 ± 18.468 (25)		.0009
CASA-Q sputum symptoms	0	Observed value	IND/GLY/MF	68.7 ± 21.2 (57)		
			ICS/LABA	68.8 ± 21.8 (52)		
			FF/VI	66.049 ± 20.139 (27)		
			BUD/FM	71.666 ± 23.448 (25)		
	4	Observed value	IND/GLY/MF	72.2 ± 24.3 (57)		
			ICS/LABA	76.8 ± 20.2 (52)		
		Change	IND/GLY/MF	3.5 ± 18.3 (57)		
			ICS/LABA	8.0 ± 18.2 (52)	.2011	
	8	Observed value	IND/GLY/MF	77.4 ± 19.6 (55)		
			ICS/LABA	77.7 ± 17.6 (52)		
			FF/VI	75.926 ± 20.193 (27)		
			BUD/FM	79.666 ± 14.449 (25)		
		Change	IND/GLY/MF	8.3 ± 17.7 (55)		.0010
			ICS/LABA	9.0 ± 18.5 (52)	.8553	
			FF/VI	9.877 ± 22.172 (27)		.0288
			BUD/FM	8.000 ± 13.921 (25)		.0084
CASA-Q sputum impact	0	Observed value	IND/GLY/MF	82.9 ± 15.7 (57)		
			ICS/LABA	83.7 ± 16.0 (52)		
			FF/VI	82.716 ± 16.371 (27)		
			BUD/FM	84.834 ± 15.768 (25)		
	4	Observed value	IND/GLY/MF	86.8 ± 15.3 (57)		
			ICS/LABA	89.6 ± 14.4 (52)		
		Change	IND/GLY/MF	3.9 ± 15.4 (57)		
			ICS/LABA	5.8 ± 10.7 (52)	.4352	
	8	Observed value	IND/GLY/MF	89.4 ± 13.4 (55)		
			ICS/LABA	89.7 ± 14.3 (52)		
			FF/VI	88.117 ± 15.663 (27)		
			BUD/FM	91.332 ± 12.725 (25)		
		Change	IND/GLY/MF	6.1 ± 16.5 (55)		.0088
			ICS/LABA	5.9 ± 14.0 (52)	.9647	
			FF/VI	5.401 ± 16.212 (27)		.0953
			BUD/FM	6.499 ± 11.352 (25)		.0086
Feno (ppb)	0	Observed value	IND/GLY/MF	25.2 ± 25.4 (57)		
			ICS/LABA	31.1 ± 39.1 (52)		
			FF/VI	31.407 ± 26.024 (27)		
			BUD/FM	30.680 ± 50.077 (25)		
	8	Observed value	IND/GLY/MF	28.7 ± 24.9 (54)		
			ICS/LABA	25.6 ± 22.6 (52)		
			FF/VI	24.604 ± 14.007 (27)		
			BUD/FM	26.760 ± 29.543 (25)		
		Change	IND/GLY/MF	3.1 ± 17.3 (54)		.1894
			ICS/LABA	−5.4 ± 20.6 (52)	.0230	
			FF/VI	−6.804 ± 17.801 (27)		.0577
			BUD/FM	−3.920 ± 23.532 (25)		.4131
Eosinophils (/μL)	0	Observed value	IND/GLY/MF	231.3 ± 221.2 (56)		
			ICS/LABA	292.3 ± 259.9 (52)		
			FF/VI	327.4 ± 298.2 (27)		
			BUD/FM	254.4 ± 210.4 (25)		
	4	Observed value	IND/GLY/MF	230.2 ± 209.9 (56)		
			ICS/LABA	233.5 ± 252.8 (52)		
		Change	IND/GLY/MF	−1.8 ± 141.3 (55)		
			ICS/LABA	−58.8 ± 131.4 (52)	.0328	
	8	Observed value	IND/GLY/MF	242.2 ± 210.0 (55)		
			ICS/LABA	237.5 ± 265.7 (52)		
			FF/VI	311.9 ± 339.6 (27)		
			BUD/FM	157.2 ± 110.4 (25)		
		Change	IND/GLY/MF	9.3 ± 118.6 (54)		.5685
			ICS/LABA	−54.8 ± 132.5 (52)	.0101	
			FF/VI	−15.6 ± 115.6 (27)		.4906
			BUD/FM	−97.2 ± 138.6 (25)		.0018
Neutrophils (/μL)	0	Observed value	IND/GLY/MF	4361.4 ± 1532.5 (56)		
			ICS/LABA	3822.9 ± 1345.1 (52)		
			FF/VI	3520.0 ± 1258.5 (27)		
			BUD/FM	4150.0 ± 1383.5 (25)		
	4	Observed value	IND/GLY/MF	4040.5 ± 1355.9 (56)		
			ICS/LABA	3962.9 ± 1325.8 (52)		
		Change	IND/GLY/MF	−362.5 ± 1380.2 (55)		
			ICS/LABA	140.0 ± 1246.0 (52)	.0504	
	8	Observed value	IND/GLY/MF	4050.2 ± 1215.2 (55)		
			ICS/LABA	4079.4 ± 1257.1 (52)		
			FF/VI	3726.7 ± 990.7 (27)		
			BUD/FM	4460.4 ± 1415.5 (25)		
		Change	IND/GLY/MF	−384.4 ± 1395.8 (54)		.0480
			ICS/LABA	256.5 ± 1244.3 (52)	.0141	
			FF/VI	206.7 ± 1031.0 (27)		.3072
			BUD/FM	310.4 ± 1460.4 (25)		.2985

∗ Comparison between IND/GLY/MF and ICS/LABA.

† Comparison between baseline and week 8.

### Capsaicin cough challenge and cough monitor tests

Capsaicin cough receptor sensitivity tests and VitaloJAK cough monitor tests showed a C2 increase from baseline to week 8 of 1.302 ± 4.444 μmol, a slight decrease in C5 of −0.265 ± 5.343 μmol, and a cough frequency reduction of −12.33 ± 17.44 coughs per hour (−11.90 ± 11.59 while awake and −13.19 ± 30.72 while asleep) in the IND/GLY/MF group.

### Safety results

The incidence of AEs was 32.2% in the IND/GLY/MF group and 22.0% in the ICS/LABA group, and the incidence of adverse drug reactions was 10.2% in the IND/GLY/MF group and 5.1% in the ICS/LABA group (Table VI). No serious or severe AEs were observed in the study. The incidence of AEs assessed as moderate in severity was 8.5% in the IND/GLY/MF group and included asthma (3.4%) and retinal degeneration, dysphonia, and pruritus (1.7% each). Moderate events in the ICS/LABA group were hypertension, asthma (asthma exacerbation), abdominal pain, and nausea (1.7% each). All other AEs were assessed as mild. The adverse drug reactions in the IND/GLY/MF group were thirst and dysphonia in 2 patients each, and oral candidiasis and oropharyngeal pain in 1 patient each. The adverse drug reactions in the ICS/LABA group were tremor, stomatitis, and muscle spasms in 1 patient each. The only adverse drug reaction assessed as moderate was a single case of dysphonia (1.7%) in the IND/GLY/MF group; no such adverse reaction occurred in the ICS/LABA group. AEs stratified by patients’ demographic and medical characteristics are presented in Table E1 for the IND/GLY/MF group and Table E2 for the ICS/LABA group, both available in the Online Repository available at www.jaci-global.org.

**Table VI:** Incidence of AEs

System organ class	AE	IND/GLY/MF (n = 59)			ICS/LABA (n = 59)		
		No. of patients	No. of AEs	Incidence rate, 95% CI	No. of patients	No. of AEs	Incidence rate, 95% CI
All	All	19	22	32.2%, 20.3-44.1	13	16	22.0%, 11.5-32.6
Infections and infestations	—	7	8	11.9%, 3.6-20.1	3	3	5.1%, 0-10.7
	Bronchitis	5	5	8.5%, 1.4-15.6	—	—	—
	Acute sinusitis	1	1	1.7%, 0-5.0	—	—	—
	Gingivitis	1	1	1.7%, 0-5.0	—	—	—
	Influenza	—	—	—	1	1	1.7%, 0-5.0
	Nasopharyngitis	—	—	—	1	1	1.7%, 0-5.0
	Oral candidiasis	1	1	1.7%, 0-5.0	—	—	—
	COVID-19	—	—	—	1	1	1.7%, 0-5.0
Psychiatric disorders	—	—	—	—	1	1	1.7%, 0-5.0
	Insomnia	—	—	—	1	1	1.7%, 0-5.0
Nervous system disorders	—	—	—	—	3	3	5.1%, 0-10.7
	Dizziness	—	—	—	1	1	1.7%, 0-5.0
	Headache	—	—	—	1	1	1.7%, 0-5.0
	Tremor	—	—	—	1	1	1.7%, 0-5.0
Eye disorders	—	1	1	1.7%, 0-5.0	—	—	—
	Retinal degeneration	1	1	1.7%, 0-5.0	—	—	—
Vascular disorders	—	—	—	—	1	1	1.7%, 0-5.0
	Hypertension	—	—	—	1	1	1.7%, 0-5.0
Respiratory, thoracic, and mediastinal disorders	—	6	6	10.2%, 2.5-17.9	1	1	1.7%, 0-5.0
	Asthma	3	3	5.1%, 0-10.7	1	1	1.7%, 0-5.0
	Dysphonia	2	2	3.4%, 0-8.0	—	—	—
	Oropharyngeal pain	1	1	1.7%, 0-5.0	—	—	—
Gastrointestinal disorders	—	—	—	—	2	3	3.4%, 0-8.0
	Abdominal pain	—	—	—	1	1	1.7%, 0-5.0
	Nausea	—	—	—	1	1	1.7%, 0-5.0
	Stomatitis	—	—	—	1	1	1.7%, 0-5.0
Skin and subcutaneous tissue disorders	—	1	2	1.7%, 0-5.0	—	—	—
	Pruritus	1	2	1.7%, 0-5.0	—	—	—
Musculoskeletal and connective tissue disorders	—	1	1	1.7%, 0-5.0	2	2	3.4%, 0-8.0
	Back pain	1	1	1.7%, 0-5.0	—	—	—
	Muscle spasms	—	—	—	1	1	1.7%, 0-5.0
	Sjögren syndrome	—	—	—	1	1	1.7%, 0-5.0
General disorders and administration-site conditions	—	2	3	3.4%, 0-8.0	1	2	1.7%, 0-5.0
	Thirst	2	2	3.4%, 0-8.0	—	—	—
	Facial pain	1	1	1.7%, 0-5.0	—	—	—
	Pyrexia	—	—	—	1	1	1.7%, 0-5.0
	Vaccination site bruising	—	—	—	1	1	1.7%, 0-5.0
Injury, poisoning, and procedural complications	—	1	1	1.7%, 0-5.0	—	—	—
	Heat illness	1	1	1.7%, 0-5.0	—	—	—
System organ class of all adverse drug reactions	All adverse drug reactions	6	6	10.2%, 2.5-17.9	3	3	5.1%, 0.0-10.7
Infections and infestations	—	1	1	1.7%, 0.0-5.0	—	—	—
	Oral candidiasis	1	1	1.7%, 0.0-5.0	—	—	—
Nervous system disorders	—	—	—	—	1	1	1.7%, 0.0-5.0
	Tremor	—	—	—	1	1	1.7%, 0.0-5.0
Respiratory, thoracic and mediastinal disorders	—	3	3	5.1%, 0.0-10.7	—	—	—
	Dysphonia	2	2	3.4%, 0.0-8.0	—	—	—
	Oropharyngeal pain	1	1	1.7%, 0.0-5.0	—	—	—
Gastrointestinal disorders	—	—	—	—	1	1	1.7%, 0.0-5.0
	Stomatitis	—	—	—	1	1	1.7%, 0.0-5.0
Musculoskeletal and connective tissue disorders	—	—	—	—	1	1	1.7%, 0.0-5.0
	Muscle spasms	—	—	—	1	1	1.7%, 0.0-5.0
General disorders and administration site conditions	—	2	2	3.4%, 0.0-8.0	—	—	—
	Thirst	2	2	3.4%, 0.0-8.0	—	—	—

*CI,* Confidence interval; *COVID-19,* coronavirus disease 2019.

No new safety signal was observed for the products used in this study.

## Discussion

Even though the efficacy of IND/GLY/MF (LABA/LAMA/ICS) compared to ICS/LABA has been established in previous studies,^16^, ^17^, ^18^, ^19^ this randomized study was the first to compare the effects of medium-dose IND/GLY/MF versus high-dose ICS/LABA on coughing in asthma patients whose cough had been resistant to medium-dose ICS/LABA treatment. Both groups showed improvement in cough symptoms and quality of life after switching to the study treatment, but the medium-dose IND/GLY/MF group was not superior to the high-dose ICS/LABA group. Several other secondary end points showed significant improvement in multiple parameters in the high-dose ICS/LABA group versus the medium-dose IND/GLY/MF group at week 4, but no difference was observed at week 8. This initial difference is considered to be due to the anti-inflammatory effect of high-dose ICS at the early stage of dose increase in the high-dose ICS/LABA group, while the medium-dose IND/GLY/MF group produced a slower sustained antitussive effect by week 8.

There are several possible reasons why this study did not show superiority of medium-dose IND/GLY/MF over high-dose ICS/LABA. First, this may be due to the influence of patient characteristics, in particular the high Feno value, which is used to assess type 2 airway inflammation, and which was higher at baseline in the ICS/LABA group. This points to an enrollment of patients in whom type 2 inflammation was not sufficiently suppressed. Lee et al^27^ showed that patients with a Feno > 25 ppb achieved the minimal clinically important change in LCQ score of ≥1.3 from baseline after treatment with high doses of ICS, showing a potential for a response. Second, the proportion of patients with moderate persistent asthma was higher in the medium-dose IND/GLY/MF group, while more patients had relatively mild asthma in the high-dose ICS/LABA group (Table I). Third, the switch from medium-dose FF/VI to high-dose BUD/FM, which occurred in 80% of patients in the BUD/FM group, may have contributed to the response to treatment in previously treatment-refractory patients simply by switching the ICS.

Another finding of note was that the blood neutrophil count significantly decreased in the medium-dose IND/GLY/MF group compared to the high-dose ICS/LABA group at week 8, suggesting a potential anti-inflammatory effect of medium-dose IND/GLY/MF. There is a report that tiotropium decreased blood neutrophil count in a single-group analysis,^14^ but our study is the first to confirm the effect in a comparative clinical setting. The observed neutrophil count decrease in this study might indicate the next clinical question, which is the effectiveness of LABA/LAMA/ICS combinations in patients with neutrophilic asthma, especially because the efficacy of LAMA against neutrophilic inflammation has already been reported in nonclinical studies.^28^ In the LABA/ICS group, there was an increase in neutrophil count, which was not statistically significant and which may have been caused by the switch of 80% patients in this group from medium-dose ICS to high-dose ICS as a result of the inhibition of apoptosis of neutrophils by glucocorticoids, which may sometimes cause proinflammatory reactions.^29^^,^^30^ In contrast, the finding that the blood eosinophil count, as well as Feno, significantly decreased in the ICS/LABA group compared to the IND/GLY/MF group points to a better effectiveness of ICS/LABA in asthma with type 2 inflammation.

A capsaicin cough receptor sensitivity test was also planned for this study, but it was optional because it could only performed in a limited number of institutions. As a result, only a small number of patients were tested (6 in the IND/GLY/MF group and 1 in the ICS/LABA group). Though the results in both tests apparently did not show a significant difference from baseline to week 8 in the IND/GLY/MF group, there was a trend of reducing cough after 8 weeks, with a C5 decrease and simultaneous C2 increase, plus a reduction in cough frequency. However, the observation that cough frequency decreased with IND/GLY/MF suggests the possibility of a significant improvement in cough in the long term, which should be investigated in further studies.

This study was designed to show superiority of IND/GLY/MF medium-dose over high-dose ICS/LABA (FF/VI or BUD/FM) for the primary end point, but the design was not chosen to demonstrate noninferiority between the treatments for any of the tested parameters. It was conducted under actual clinical settings and may have limitations due to the open-label design (eg, bias from patient expectations of a new treatment), the low patient number, and the short evaluation period of 8 weeks. Also, the period of 1 month before the study, in which patients had to be treated unsuccessfully for their cough, may have been too short. The percentage of patients who had received medium-dose FF/VI as prior treatment was approximately 80% in both groups, which was higher than expected, and the switch to a high-dose of ICS may have affected the evaluation of cough symptoms. Some patients randomized to high-dose ICS/LABA remained on their familiar inhaler device, while all patients randomized to medium-dose IND/GLY/MF were switched to a new device, which was provided with inhalation instructions, possibly resulting in differences in inhalation techniques. The adherence to the treatment was not assessed, though this was a limitation affecting both treatment groups. In addition, the clinical setting of this study, which included asthma patients with refractory cough, mirrors a real-life challenge when treating asthma. The study results present an alternative treatment option to high-dose ICS/LABA, with comparable outcomes for bronchodilatation, cough and sputum symptoms, and a potential additional anti-inflammatory effect, but without the increased risk of adverse effects from high ICS doses.

Regarding safety, no clinically significant adverse drug reactions occurred in either group, but long-term therapy with high-dose ICS is known to be associated with a risk of adverse drug reactions such as hypothalamic–pituitary–adrenal axis suppression, reduction in growth velocity, osteoporosis, diabetes, and respiratory infections.^31^

In conclusion, cough symptoms improved comparably between medium-dose IND/GLY/MF and high-dose ICS/LABA combinations; however, superiority of medium-dose IND/GLY/MF could not be demonstrated. Moreover, this study was the first to show a decrease in neutrophil count with LABA/LAMA/ICS in a clinical study. Even though no differences in safety were seen during the study’s short duration, the addition of LAMA to the treatment regimen may be a preferable alternative to increasing the ICS dose, with the known adverse effects for long-term receipt in asthma patients with refractory cough, who are currently treated long term with high-dose ICS/LABA.

**Clinical implication**

Medium-dose IND/GLY/MF showed comparable improvements of refractory cough in asthma patients as high-dose ICS/LABA and may be a preferable alternative for long-term treatment because of the lower dose of ICS.

## Disclosure statement

Funded by 10.13039/100008792Novartis Pharma K.K., Japan. Employees of Novartis Pharma were involved in the study design, analysis plan, and interpretation of the data.

Data sharing statement: Data to support the findings of this study are available from 10.13039/501100008883Nagoya City University, but availability of the data is restricted and can only be granted under license, and cannot be published by the licensee. Data are, however, available from the authors on reasonable request and with the permission of Novartis Pharma.

Disclosure of potential conflict of interest: A. Niimi has received speaker fees from Novartis Pharma, AstraZeneca, GlaxoSmithKline, and Boehringer. H. Ohbayashi has received speaker fees from GlaxoSmithKline, and has served as a representative of Kracie. A. Kiyosue has received speaker fees from Novartis Pharma. Y. Fukushima has received study materials for this report and was involved in medical writing. T. Tochigi had received fees as a speaker and for expert testimony in a keynote lecture from GlaxoSmithKline, and support for attending meetings from Novartis Pharma and GlaxoSmithKline. T. Tsuji and T. Mikami are employees of Novartis Pharma. Y. Kanemitsu has received funding from Novartis Pharma for this report, research grants from MSD/MSD life foundations; speaker fees from GlaxoSmithKline, AstraZeneca, Sanofi, Kyorin Pharmaceutical, Novartis Pharma, and Zeria Pharmaceutical, and support for attending meetings from Sanofi and GlaxoSmithKline. T. Tajiri has received lecturer fees from Sanofi. J. Hara has received speaker fees from AstraZeneca, GlaxoSmithKline, and Kyorin Pharmaceutical. The rest of the authors declare that they have no relevant conflicts of interest.

## Supplementary data

Supplementary Fig E1

Supplementary Fig E2

Supplementary Fig E3

Supplementary Table E1

Supplementary Table E2
