# Supplementary material for: Effectiveness of indacaterol/glycopyrronium/mometasone for refractory asthmatic cough after switching from inhaled corticosteroid/long-acting β2-agonist therapy
Source: J Allergy Clin Immunol Glob. 2025 Sep 8;4(4):100567. doi: 10.1016/j.jacig.2025.100567 (PMC12528903; doi:10.1016/j.jacig.2025.100567)
Supplement: Supplementary Fig E1 [file mmc1.pptx]

## Slide 1
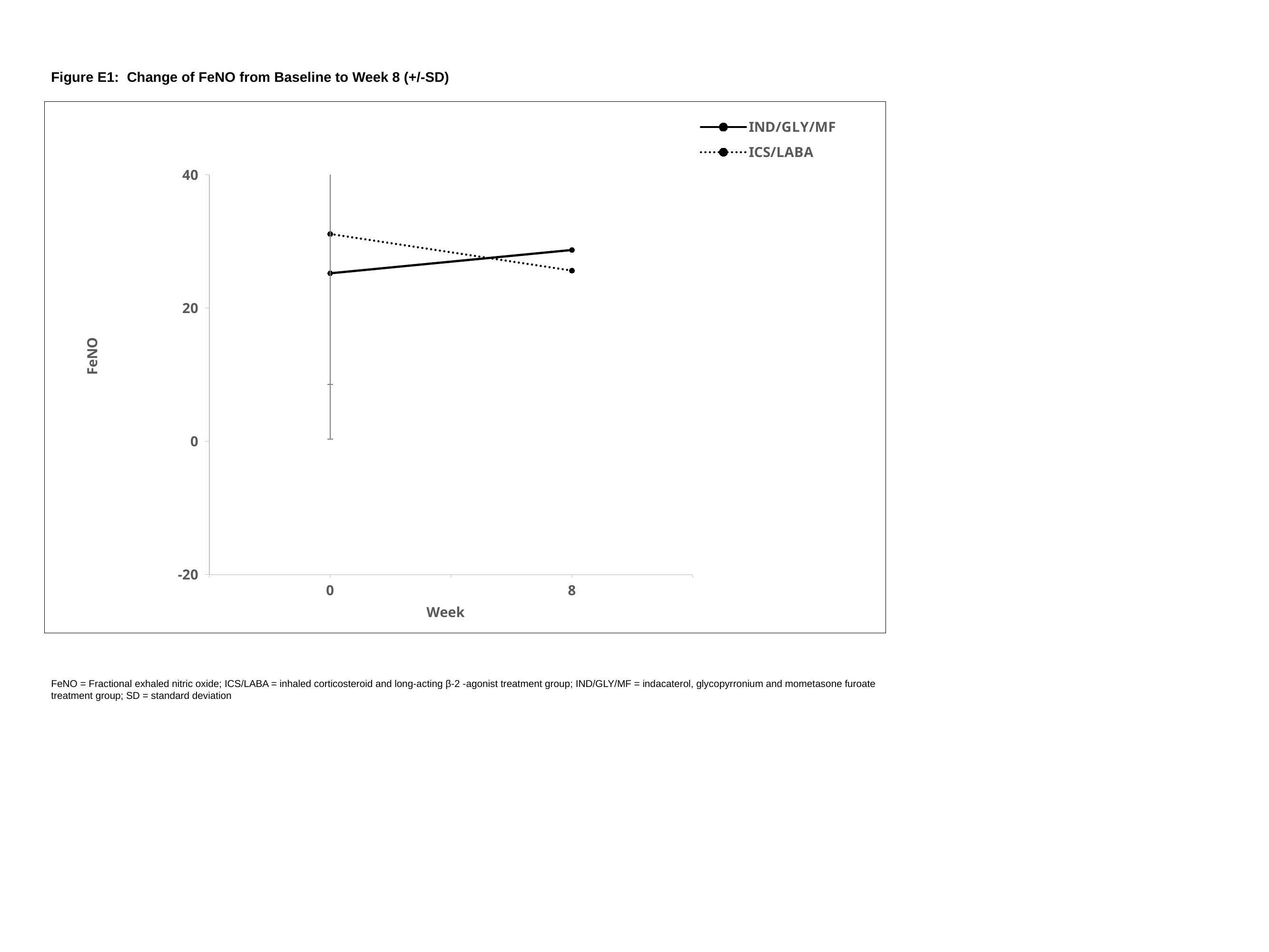

Figure E1:  Change of FeNO from Baseline to Week 8 (+/-SD)
### Chart
| Category | IND/GLY/MF | ICS/LABA |
|---|---|---|
| 0 | 25.2 | 31.1 |
| 8 | 28.7 | 25.6 |FeNO = Fractional exhaled nitric oxide; ICS/LABA = inhaled corticosteroid and long-acting β-2 -agonist treatment group; IND/GLY/MF = indacaterol, glycopyrronium and mometasone furoate treatment group; SD = standard deviation
