# Supplementary material for: Effectiveness of indacaterol/glycopyrronium/mometasone for refractory asthmatic cough after switching from inhaled corticosteroid/long-acting β2-agonist therapy
Source: J Allergy Clin Immunol Glob. 2025 Sep 8;4(4):100567. doi: 10.1016/j.jacig.2025.100567 (PMC12528903; doi:10.1016/j.jacig.2025.100567)
Supplement: Supplementary Fig E3 [file mmc3.pptx]

## Slide 1
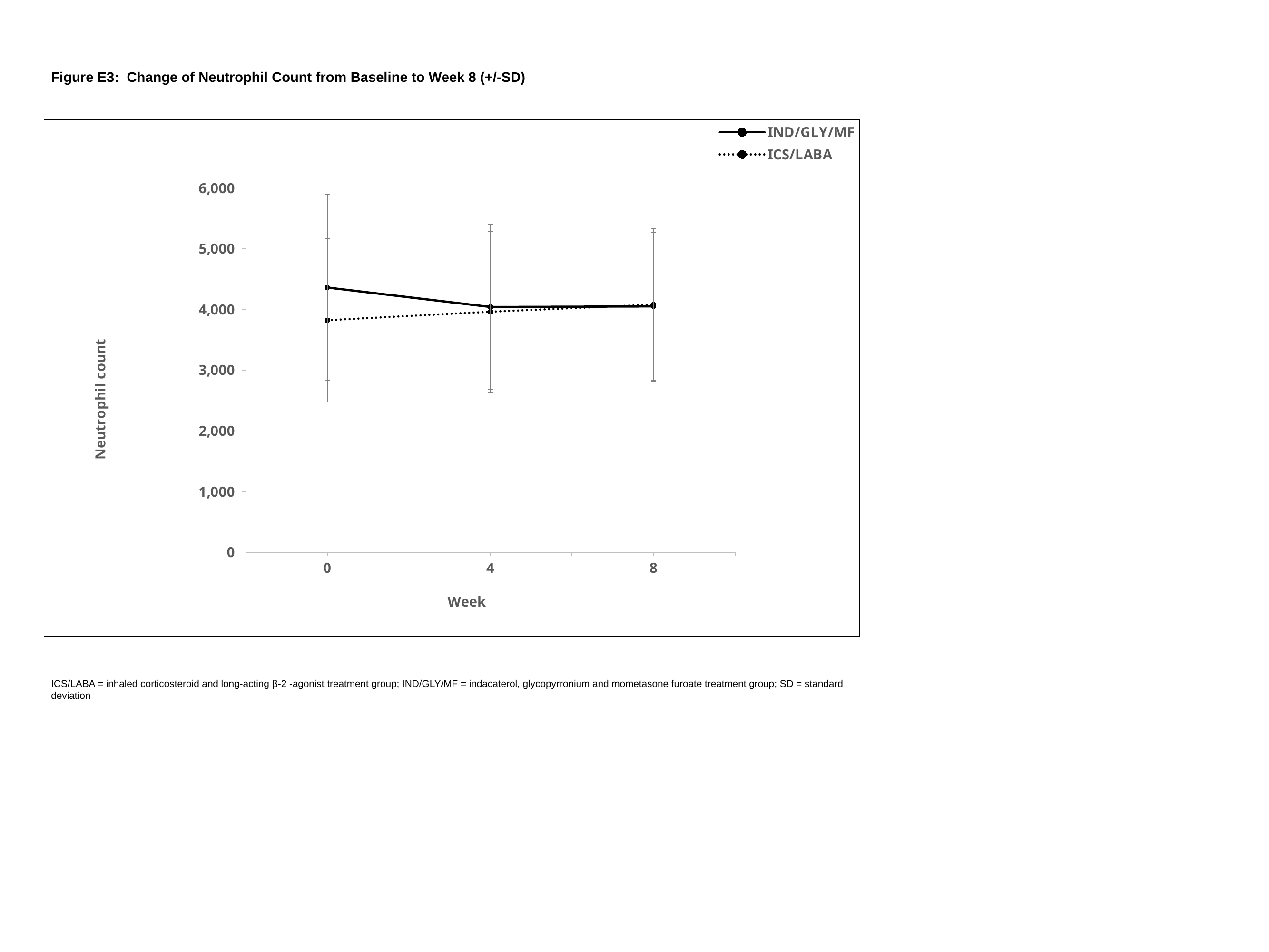

Figure E3:  Change of Neutrophil Count from Baseline to Week 8 (+/-SD)
### Chart
| Category | IND/GLY/MF | ICS/LABA |
|---|---|---|
| 0 | 4361.4 | 3822.9 |
| 4 | 4040.5 | 3962.9 |
| 8 | 4050.2 | 4079.4 |ICS/LABA = inhaled corticosteroid and long-acting β-2 -agonist treatment group; IND/GLY/MF = indacaterol, glycopyrronium and mometasone furoate treatment group; SD = standard deviation
