# Supplementary material for: Effectiveness of indacaterol/glycopyrronium/mometasone for refractory asthmatic cough after switching from inhaled corticosteroid/long-acting β2-agonist therapy
Source: J Allergy Clin Immunol Glob. 2025 Sep 8;4(4):100567. doi: 10.1016/j.jacig.2025.100567 (PMC12528903; doi:10.1016/j.jacig.2025.100567)
Supplement: Supplementary Table E1 [file mmc4.docx]

**Table E1: Stratified Tabulation of AEs (IND/GLY/MF)**

| **Parameter** | **Category** | **Number of patients in the category** | **Number of patients with AEs** | **Incidence rate,**  **95%CI** |
| --- | --- | --- | --- | --- |
| **Gender** | Male | N=18 | 5 | 27.8%, 7.1 - 48.5% |
|  | Female | N=41 | 14 | 34.1%, 19.6 - 48.7% |
| **Age** | min~median | N=29 | 10 | 34.5%, 17.2 - 51.8% |
|  | median~max | N=30 | 9 | 30.0%, 13.6 - 46.4% |
| **BMI** | min~median | N=27 | 8 | 29.6%, 12.4 - 46.9% |
|  | median~max | N=32 | 11 | 34.4%, 17.9 - 50.8% |
| **Smoking history** | No | N=52 | 15 | 28.8%, 16.5 - 41.2% |
|  | Yes | N=7 | 4 | 57.1%, 20.5 - 93.8% |
| **Complications** | No | N=9 | 1 | 11.1%, 0.0 - 31.6% |
|  | Yes | N=50 | 18 | 36.0%, 22.7 - 49.3% |
| **Medical history** | No | N=43 | 14 | 32.6%, 18.6 - 46.6% |
|  | Yes | N=16 | 5 | 31.3%, 8.5 - 54.0% |
| **Duration of asthma** | min~median | N=33 | 12 | 36.4%, 20.0 - 52.8% |
|  | median~max | N=26 | 7 | 26.9%, 9.9 - 44.0% |
| **Severity of asthma** | Mild persistent | N=8 | 2 | 25.0%, 0.0 - 55.0% |
|  | Moderate persistent | N=50 | 17 | 34.0%, 20.9 - 47.1% |
|  | Severe persistent | N=1 | 0 | 0.0%, 0.0 - 0.0% |
|  | Most severe persistent | N=0 | - | - |
| **History of childhood asthma** | No | N=55 | 18 | 32.7%, 20.3 - 45.1% |
|  | Yes | N=4 | 1 | 25.0%, 0.0 - 67.4% |
| **Family history of asthma** | No | N=41 | 14 | 34.1%, 19.6 - 48.7% |
|  | Yes | N=18 | 5 | 27.8%, 7.1 - 48.5% |
| **Concomitant medications for asthma** | No | N=32 | 7 | 21.9%, 7.6 - 36.2% |
|  | Yes | N=27 | 12 | 44.4%, 25.7 - 63.2% |
| **J-LCQ  (total score)** | min~median | N=28 | 11 | 39.3%, 21.2 - 57.4% |
|  | median~max | N=31 | 8 | 25.8%, 10.4 - 41.2% |
| **Cough VAS score  (while awake)** | min~median | N=30 | 6 | 20.0%, 5.7 - 34.3% |
|  | median~max | N=29 | 13 | 44.8%, 26.7 - 62.9% |
| **Cough VAS score  (during sleep)** | min~median | N=28 | 7 | 25.0%, 9.0 - 41.0% |
|  | median~max | N=31 | 12 | 38.7%, 21.6 - 55.9% |
| **FEV_1_** | min~median | N=30 | 11 | 36.7%, 19.4 - 53.9% |
|  | median~max | N=29 | 8 | 27.6%, 11.3 - 43.9% |
| **FVC** | min~median | N=30 | 11 | 36.7%, 19.4 - 53.9% |
|  | median~max | N=29 | 8 | 27.6%, 11.3 - 43.9% |
| **FEF_25-75_** | min~median | N=28 | 9 | 32.1%, 14.8 - 49.4% |
|  | median~max | N=31 | 10 | 32.3%, 15.8 - 48.7% |
| **%FEV_1_** | min~median | N=31 | 10 | 32.3%, 15.8 - 48.7% |
|  | median~max | N=28 | 9 | 32.1%, 14.8 - 49.4% |
| **FEV_1_%** | min~median | N=31 | 10 | 32.3%, 15.8 - 48.7% |
|  | median~max | N=28 | 9 | 32.1%, 14.8 - 49.4% |
| **FeNO** | min~median | N=30 | 12 | 40.0%, 22.5 - 57.5% |
|  | median~max | N=29 | 7 | 24.1%, 8.6 - 39.7% |
| **Eosinophils** | min~median | N=35 | 10 | 28.6%, 13.6 - 43.5% |
|  | median~max | N=23 | 8 | 34.8%, 15.3 - 54.2% |
| **Neutrophils** | min~median | N=23 | 7 | 30.4%, 11.6 - 49.2% |
|  | median~max | N=35 | 11 | 31.4%, 16.0 - 46.8% |
| **ACQ-6  (total score)** | min~median | N=22 | 3 | 13.6%, 0.0 - 28.0% |
|  | median~max | N=37 | 16 | 43.2%, 27.3 - 59.2% |
| **CASA-Q  (total score)** | min~median | N=30 | 13 | 43.3%, 25.6 - 61.1% |
|  | median~max | N=29 | 6 | 20.7%, 5.9 - 35.4% |
