# Supplementary material for: Effectiveness of indacaterol/glycopyrronium/mometasone for refractory asthmatic cough after switching from inhaled corticosteroid/long-acting β2-agonist therapy
Source: J Allergy Clin Immunol Glob. 2025 Sep 8;4(4):100567. doi: 10.1016/j.jacig.2025.100567 (PMC12528903; doi:10.1016/j.jacig.2025.100567)
Supplement: Supplementary Table E2 [file mmc5.docx]

**Table E2: Stratified Tabulation of AEs (ICS/LABA)**

| **Parameter** | **Category** | **Number of patients in the category** | **Number of patients with AEs** | **Incidence rate, 95%CI** |
| --- | --- | --- | --- | --- |
| **Gender** | Male | N=20 | 2 | 10.0%, 0.0 - 23.1% |
|  | Female | N=39 | 11 | 28.2%, 14.1 - 42.3% |
| **Age** | min~median | N=28 | 6 | 21.4%, 6.2 - 36.6% |
|  | median~max | N=31 | 7 | 22.6%, 7.9 - 37.3% |
| **BMI** | min~median | N=31 | 8 | 25.8%, 10.4 - 41.2% |
|  | median~max | N=28 | 5 | 17.9%, 3.7 - 32.0% |
| **Smoking history** | No | N=49 | 12 | 24.5%, 12.4 - 36.5% |
|  | Yes | N=10 | 1 | 10.0%, 0.0 - 28.6% |
| **Complications** | No | N=12 | 1 | 8.3%, 0.0 - 24.0% |
|  | Yes | N=47 | 12 | 25.5%, 13.1 - 38.0% |
| **Medical history** | No | N=45 | 7 | 15.6%, 5.0 - 26.1% |
|  | Yes | N=14 | 6 | 42.9%, 16.9 - 68.8% |
| **Duration of asthma** | min~median | N=26 | 6 | 23.1%, 6.9 - 39.3% |
|  | median~max | N=33 | 7 | 21.2%, 7.3 - 35.2% |
| **Severity of asthma** | Mild persistent | N=15 | 2 | 13.3%, 0.0 - 30.5% |
|  | Moderate persistent | N=40 | 11 | 27.5%, 13.7 - 41.3% |
|  | Severe persistent | N=4 | 0 | 0.0%, 0.0 - 0.0% |
|  | Most severe persistent | N=0 |  |  |
| **History of childhood asthma** | No | N=45 | 11 | 24.4%, 11.9 - 37.0% |
|  | Yes | N=14 | 2 | 14.3%, 0.0 - 32.6% |
| **Family history of asthma** | No | N=38 | 7 | 18.4%, 6.1 - 30.7% |
|  | Yes | N=21 | 6 | 28.6%, 9.2 - 47.9% |
| **Concomitant medications for asthma** | No | N=35 | 6 | 17.1%, 4.7 - 29.6% |
|  | Yes | N=24 | 7 | 29.2%, 11.0 - 47.4% |
| **J-LCQ  (total score)** | min~median | N=31 | 6 | 19.4%, 5.4 - 33.3% |
|  | median~max | N=28 | 7 | 25.0%, 9.0 - 41.0% |
| **Cough VAS score  (while awake)** | min~median | N=29 | 8 | 27.6%, 11.3 - 43.9% |
|  | median~max | N=30 | 5 | 16.7%, 3.3 - 30.0% |
| **Cough VAS score  (during sleep)** | min~median | N=31 | 7 | 22.6%, 7.9 - 37.3% |
|  | median~max | N=28 | 6 | 21.4%, 6.2 - 36.6% |
| **FEV_1_** | min~median | N=28 | 6 | 21.4%, 6.2 - 36.6% |
|  | median~max | N=31 | 7 | 22.6%, 7.9 - 37.3% |
| **FVC** | min~median | N=28 | 6 | 21.4%, 6.2 - 36.6% |
|  | median~max | N=31 | 7 | 22.6%, 7.9 - 37.3% |
| **FEF_25-75_** | min~median | N=29 | 6 | 20.7%, 5.9 - 35.4% |
|  | median~max | N=30 | 7 | 23.3%, 8.2 - 38.5% |
| **%FEV_1_** | min~median | N=28 | 5 | 17.9%, 3.7 - 32.0% |
|  | median~max | N=31 | 8 | 25.8%, 10.4 - 41.2% |
| **FEV_1_%** | min~median | N=28 | 4 | 14.3%, 1.3 - 27.2% |
|  | median~max | N=31 | 9 | 29.0%, 13.1 - 45.0% |
| **FeNO** | min~median | N=28 | 7 | 25.0%, 9.0 - 41.0% |
|  | median~max | N=31 | 6 | 19.4%, 5.4 - 33.3% |
| **Eosinophils** | min~median | N=22 | 3 | 13.6%, 0.0 - 28.0% |
|  | median~max | N=37 | 10 | 27.0%, 12.7 - 41.3% |
| **Neutrophils** | min~median | N=35 | 9 | 25.7%, 11.2 - 40.2% |
|  | median~max | N=24 | 4 | 16.7%, 1.8 - 31.6% |
| **ACQ-6  (total score)** | min~median | N=29 | 8 | 27.6%, 11.3 - 43.9% |
|  | median~max | N=30 | 5 | 16.7%, 3.3 - 30.0% |
| **CASA-Q  (total score)** | min~median | N=29 | 7 | 24.1%, 8.6 - 39.7% |
|  | median~max | N=30 | 6 | 20.0%, 5.7 - 34.3% |
