# Supplementary material for: Effectiveness of indacaterol/glycopyrronium/mometasone for refractory asthmatic cough after switching from inhaled corticosteroid/long-acting β2-agonist therapy
Source: J Allergy Clin Immunol Glob. 2025 Sep 8;4(4):100567. doi: 10.1016/j.jacig.2025.100567 (PMC12528903; doi:10.1016/j.jacig.2025.100567)
Supplement: Supplementary Data [file mmc6.docx]

Supplemental Material

Figure E1: Change of FeNO from Baseline to Week 8 (+/-SD)

Figure E2: Change of Eosinophil Count from Baseline to Week 8 (+/-SD)

Figure E3: Change of Neutrophil Count from Baseline to Week 8 (+/-SD)

Table E1: Stratified tabulation of AEs (IND/GLY/MF)

Table E2: Stratified tabulation of AEs (ICS/LABA)
